# Identification and expression analysis of *Sox* family genes in echinoderms

**DOI:** 10.1186/s12864-024-10547-0

**Published:** 2024-07-01

**Authors:** Xiaojing Li, Tiangui Cao, Hui Liu, Longhai Fu, Quanchao Wang

**Affiliations:** 1 Yantai Vocational College, Yantai, 264003 China; 2 Yantai Institute of Coastal Zone Research, Chinese Academy of Sciences, Yantai, 264003 China; 3 Key Laboratory of Ecological Warning, Protection & Restoration for Bohai Sea, Ministry of Natural Resources, Qingdao, 266061 China

**Keywords:** Echinoderm, Sox, Identification, Phylogeny, Expression

## Abstract

The *Sox* gene family, a collection of transcription factors widely distributed throughout the animal kingdom, plays a crucial role in numerous developmental processes. Echinoderms occupy a pivotal position in many research fields, such as neuroscience, sex determination and differentiation, and embryonic development. However, to date, no comprehensive study has been conducted to characterize and analyze *Sox* genes in echinoderms. In the present study, the evolution and expression of *Sox* family genes across 11 echinoderms were analyzed using bioinformatics methods. The results revealed a total of 70 Sox genes, with counts ranging from 5 to 8 across different echinoderms. Phylogenetic analysis revealed that the identified *Sox* genes could be categorized into seven distinct classes: the *SoxB1* class, *SoxB2* class, *SoxC* class, *SoxD* class, *SoxE* class, *SoxF* class and *SoxH* class. Notably, the *SoxB1*, *SoxB2*, and *SoxF* genes were ubiquitously present in all the echinoderms studied, which suggests that these genes may be conserved in echinoderms. The spatiotemporal expression patterns observed for *Sox* genes in the three echinoderms indicated that various *Sox* members perform distinct functional roles. Notably, *SoxB1* is likely involved in echinoderm ovary development, while *SoxH* may play a crucial role in testis development in starfish and sea cucumber. In general, the present investigation provides a molecular foundation for exploring the *Sox* gene in echinoderms, providing a valuable resource for future phylogenetic and genomic studies.

**Supplementary Information:**

The online version contains supplementary material available at 10.1186/s12864-024-10547-0.

## Introduction

*Sox* genes are distinguished by the presence of a high-mobility group (HMG) box, which comprises 79 amino acids. Since the initial discovery of the first *Sox* family gene, *Sry*, in 1990 [1], the *Sox* gene family has grown significantly, with more than 100 members identified across a diverse range of organisms, ranging from mammals and birds to reptiles, fishes, and even insects. The *Sox* family is divided into several distinct subfamilies [2]. The *Sox* family of genes plays crucial roles in various developmental processes, including sex determination/differentiation, endoderm development, angiogenesis, chondrogenesis, neurogenesis, and cardiogenesis. The breadth and depth of their influence across these diverse biological processes underscore the significance of the *Sox* family in the intricate machinery of life.

To obtain a thorough understanding of the evolution and function of the *Sox* family, it is paramount to meticulously characterize *Sox* genes across a diverse array of phyla. Previous studies have examined *Sox* family genes in mammals, teleosts, and invertebrates, revealing significant differences among these organisms. For instance, in mammals such as mice, humans, and buffalo, a total of 20 *Sox* genes, which can be subdivided into 8 subgroups (A, B1/B2, C, D, E, F, G, and H), have been identified [3, 4]. In contrast, teleosts exhibit a much larger *Sox* gene family, with 29 members reported in *Oncorhynchus mykiss* [5], 27 genes in *Oreochromis niloticus* [6], and 26 genes in *Collichthys lucidus* [7] and *Danio rerio* [8]. On the other hand, the *Sox* gene family appears to be more conserved in invertebrates, with 7 members in *Patinopecten yessoensis* [9] and 8 members in *Drosophila melanogaster* [10]. However, despite these advances, a critical gap remains in our knowledge of *Sox* genes in echinoderms. To date, no comprehensive study has been undertaken to identify and analyze *Sox* genes within this phylogenetically distinct group of organisms.

Echinoderms, long recognized as the invertebrate sister group nearest to vertebrates, possess a unique and distinct evolutionary classification [11]. As an ancient invertebrate group, echinoderms exhibit an exceptional array of reproductive modes [12]. Due to their rich biological characteristics, echinoderms have emerged as crucial research subjects in various fields, including embryonic development, sex determination and differentiation, and regeneration biology. Notably, recent investigations have indicated a potential link between *Sox* genes and certain biological processes in echinoderms [13, 14]. Despite these intriguing findings, there has been a notable absence of research focusing on a comprehensive analysis of *Sox* family genes in echinoderms.

The primary aim of this study was to conduct a thorough analysis of the abundance and expression patterns of *Sox* genes in echinoderms. Through the decoding of numerous echinoderm genomes, genome-wide identification and expression analysis of the *Sox* gene family were performed. The outcomes of this study have the potential to aid in elucidating the evolution and potential functions of *Sox* genes in echinoderms.

## Materials and methods

### Sequence identification

The *Sox* genes in 11 echinoderms, including *Anneissia japonica*, *Acanthaster planc*, *Apostichopus japonicus*, *Asterias rubens*, *Heliocidaris erythrogramma*, *Heliocidaris tuberculate*, *Holothuria leucospilota*, *Lytechinus variegatus*, *Patiria miniate*, *Plazaster borealis*, and *Strongylocentrotus purpuratus*, were identified through a combination of HMM and BLAST search methods. Initially, the relevant files for the echinoderms were downloaded from their respective databases, and the HMG domain query (accession: PF00505) was collected from the InterPro database (https://www.ebi.ac.uk/interpro/). Subsequently, a concurrent search for SOX proteins within all the genomes was conducted using both HMMER V3.4 [15] and BLAST V2.12.0 [16] through the HMG domain. The initial E values were set at 1.0 for the HMM searches and 1 × 10^− 5^ for BLAST. Next, the candidate genes identified by both methods were merged, and any duplicate genes were eliminated. If several transcripts were annotated for a specific gene, the transcript exhibiting the longest length was chosen for further analysis. Additionally, potential SOX orthologs were screened for the conserved motif RPMNAFMVW [17]. Finally, the protein properties of the identified *Sox* genes were calculated using TBtools v2.096 [18].

### Phylogenetic analysis

Diverse sets of Sox protein sequences from various species, including humans, mice, zebrafish, tilapia, and fruit fly, were downloaded from the NCBI (Supplementary Table S1). The amino acid sequence of the HMG box of these Sox proteins, along with those identified from 11 echinoderms, were extracted utilizing the Batch SMART plug-in within TBtools v2.096 [18]. These sequences were subsequently subjected to phylogenetic analysis. MAFFT v7.525 [19] was used to generate multiple sequence alignments. Subsequently, IQTREE v2.3.1 [20] was employed to construct phylogenetic trees utilizing the specific settings of --bnni, -m MFP, -B 4000, and -T AUTO. The phylogenetic tree was then visualized through the use of an online tool (iTOL) [21].

### Conserved domain, gene structure and motif

To clarify the *Sox* gene structure and exon details, a general feature format file (GFF) was utilized. Prediction of the conserved motifs within the *Sox* genes was achieved through the application of MEME [22], with the following parameters: a ceiling of 20 motifs, a minimum motif length of 6, a maximum motif length of 50, and default settings for all remaining parameters. Both the conserved motifs and the gene structure were graphically represented using TBtools v2.096 [18]. Furthermore, the conserved domains of *Sox* genes were identified utilizing the Batch SMART plug-in within TBtools v2.096 [18], and the results were visualized through the iTOL online tool [21].

### Expression profiling of *Sox* genes in different echinoderms

To investigate the spatiotemporal expression patterns of *Sox* genes in echinoderms, publicly accessible RNA-seq data for *S. purpuratus*, *A. japonicus*, and *H. leucospilota* were retrieved from the NCBI SRA database (refer to Data availability). Subsequently, Fastp software [20], with default parameters, was used to filter the raw RNA sequencing reads. Next, the genome was indexed, and the filtered reads were aligned utilizing HISAT2 [23]. After converting the resulting Sam files to Bam files and sorting them with SAMtools [24], the TPM value for each gene was calculated by StringTie v2.2.0 [25] according to the gff file. The TPM values were categorized as follows: <2, no expression; <20, very low expression; <100, low expression; <500, moderate expression; <2500, high expression; and < 12,500, very high expression. Finally, heatmaps depicting the gene expression levels were created using the R package ggplot2 [26]. Additionally, the available single-cell sequencing data from *S. purpuratus* [27] were downloaded (GEO: GSE149221) and analyzed to better elucidate the expression pattern of the *Sox* genes during early development.

## Results

### Identification of *Sox* genes in echinoderms

A comprehensive analysis of 11 representative echinoderms yielded the discovery of a set of 70 *Sox* genes. For reference purposes, the complete amino acid sequences of these *Sox* genes are listed in Supplementary Table S2. Across the various species examined, the number of *Sox* genes observed varied from 5 to 8. Moreover, the characteristics of all the identified Sox proteins are comprehensively outlined in Table 1. The findings revealed significant differences in the biophysical properties of these Sox proteins. Specifically, the amino acid (AA) length of these proteins varies widely, ranging from 114 to 1367 residues. Similarly, the molecular weight (MW) also exhibited a broad spectrum, falling within the range of 13329.97 to 149705.86 Da. Additionally, the protein instability index (PI) values varied significantly, ranging from 5.20 to 10.70. Notably, the majority of the Sox proteins analyzed demonstrated instability indices exceeding 40, indicating their inherent instability.

**Table 1:** Protein sequence features of the identified *sox* genes in echinoderms

Species	Gene ID	AA	MW	PI	INS	AIN	GRAVY
*Acanthaster planci*	AcpSoxB1	323	35.76	9.65	47.53	47.52	-0.86
	AcpSoxB2	258	28.94	9.80	72.30	59.07	-0.70
	AcpSoxC	377	42.33	7.76	70.46	59.02	-0.97
	AcpSoxD	740	81.19	6.56	61.67	67.42	-0.70
	AcpSoxF	496	54.08	5.20	54.37	62.46	-0.65
	AcpSoxH	896	95.70	8.09	50.70	66.13	-0.56
*Anneissia japonica*	AnjSoxB1	360	38.79	9.77	51.05	54.86	-0.63
	AnjSoxB2	248	28.17	9.91	58.04	65.04	-0.66
	AnjSoxD	669	76.13	6.68	60.06	69.82	-0.84
	AnjSoxE	429	48.35	6.84	61.47	46.60	-1.04
	AnjSoxF	450	49.52	6.38	57.44	59.22	-0.71
	AnjSoxH	823	93.15	8.53	51.57	62.67	-0.85
*Apostichopus japonicus*	ApjSoxB1	355	39.16	9.65	50.85	53.15	-0.82
	ApjSoxB2	122	14.70	10.19	73.61	44.84	-1.42
	ApjSoxC	382	41.71	5.62	56.16	64.11	-0.68
	ApjSoxD	236	27.32	9.73	56.84	60.00	-0.96
	ApjSoxE	459	50.64	6.70	62.40	51.24	-0.86
	ApjSoxF	203	23.15	10.70	49.48	58.67	-0.96
	ApjSoxH	114	13.33	10.43	44.26	43.60	-1.48
*Asterias rubens*	AsrSoxB1	319	35.24	9.72	54.23	49.94	-0.84
	AsrSoxB2	257	28.86	9.86	64.30	59.73	-0.68
	AsrSoxC	381	42.77	7.27	71.69	59.66	-0.90
	AsrSoxD	719	79.78	7.15	59.38	66.24	-0.75
	AsrSoxE	468	52.14	6.67	71.71	52.97	-0.98
	AsrSoxF	463	51.21	5.72	56.60	64.34	-0.67
	AsrSoxH	456	49.94	8.97	47.91	66.58	-0.71
*Heliocidaris erythrogramma*	HeeSoxB1	345	37.35	9.76	48.60	53.59	-0.73
	HeeSoxB2	267	29.43	9.83	52.63	59.81	-0.57
	HeeSoxC	368	40.58	6.68	59.70	59.89	-0.72
	HeeSoxD	737	81.60	6.61	65.81	69.82	-0.79
	HeeSoxE	499	55.40	7.29	70.35	50.68	-0.88
	HeeSoxF	484	53.46	5.94	60.56	58.49	-0.76
*Heliocidaris tuberculata*	HetSoxB1	345	37.42	9.76	47.16	53.01	-0.75
	HetSoxB2	267	29.43	9.83	52.63	59.81	-0.57
	HetSoxC	368	40.59	6.68	60.08	59.89	-0.72
	HetSoxD	736	81.34	6.73	64.49	69.93	-0.78
	HetSoxE1	496	55.07	7.25	70.49	50.00	-0.88
	HetSoxE2	496	55.07	7.25	70.49	50.00	-0.88
	HetSoxF	484	53.40	5.94	60.18	58.29	-0.77
*Holothuria leucospilota*	HolSoxB1	358	39.32	9.69	52.23	54.64	-0.76
	HolSoxB2	265	29.76	9.90	67.00	59.74	-0.69
	HolSoxC	393	42.94	5.66	62.45	60.31	-0.70
	HolSoxE	449	50.05	6.68	66.78	48.91	-0.92
	HolSoxF	570	63.12	6.11	55.35	70.67	-0.61
	HolSoxH	1367	149.71	9.39	51.36	61.02	-0.83
*Lytechinus variegatus*	LyvSoxB1	351	38.01	9.70	46.39	53.99	-0.74
	LyvSoxB2	267	29.40	9.83	54.47	60.19	-0.56
	LyvSoxC	369	40.71	6.72	60.23	59.46	-0.73
	LyvSoxD	723	79.79	6.60	65.02	67.55	-0.82
	LyvSoxE	499	55.48	7.25	69.81	49.68	-0.90
	LyvSoxF	485	53.48	5.91	56.86	59.20	-0.76
*Patiria miniata*	PamSoxB1	323	35.74	9.65	50.24	46.32	-0.87
	PamSoxB2	258	28.94	9.80	71.84	60.19	-0.71
	PamSoxC	384	43.07	7.75	68.80	60.47	-0.91
	PamSoxD	716	78.36	6.26	62.48	66.27	-0.71
	PamSoxE	475	53.08	6.78	67.64	52.84	-0.97
	PamSoxF1	494	54.18	5.50	50.80	60.30	-0.68
	PamSoxF2	494	54.18	5.50	50.80	60.30	-0.68
	PamSoxH	974	105.21	5.51	59.20	63.18	-0.70
*Plazaster borealis*	PlbSoxB1	319	35.22	9.72	55.42	50.56	-0.83
	PlbSoxB2	258	28.96	9.86	64.03	60.27	-0.67
	PlbSoxE	461	51.37	6.90	73.06	53.15	-0.97
	PlbSoxF	467	51.41	5.83	53.86	63.17	-0.69
	PlbSoxH	855	91.72	7.66	51.09	66.85	-0.70
*Strongylocentrotus purpuratus*	StpSoxB1	344	37.28	9.76	47.60	53.72	-0.71
	StpSoxB2	270	29.72	9.75	50.43	58.44	-0.56
	StpSoxC	367	40.67	7.09	59.56	60.57	-0.73
	StpSoxD	736	81.30	6.78	63.07	70.46	-0.74
	StpSoxE	496	55.18	7.71	72.35	50.56	-0.88
	StpSoxF	487	53.91	6.06	59.95	59.14	-0.73

AA, amino acid length; MW, molecular weight, KD; PI, isoelectric point; INS, instability index; AIN, aliphatic index; GRAVY, grand average of hydropathy

### Phylogenetic tree of the Sox proteins from echinoderms and other animals

**Fig. 1:**
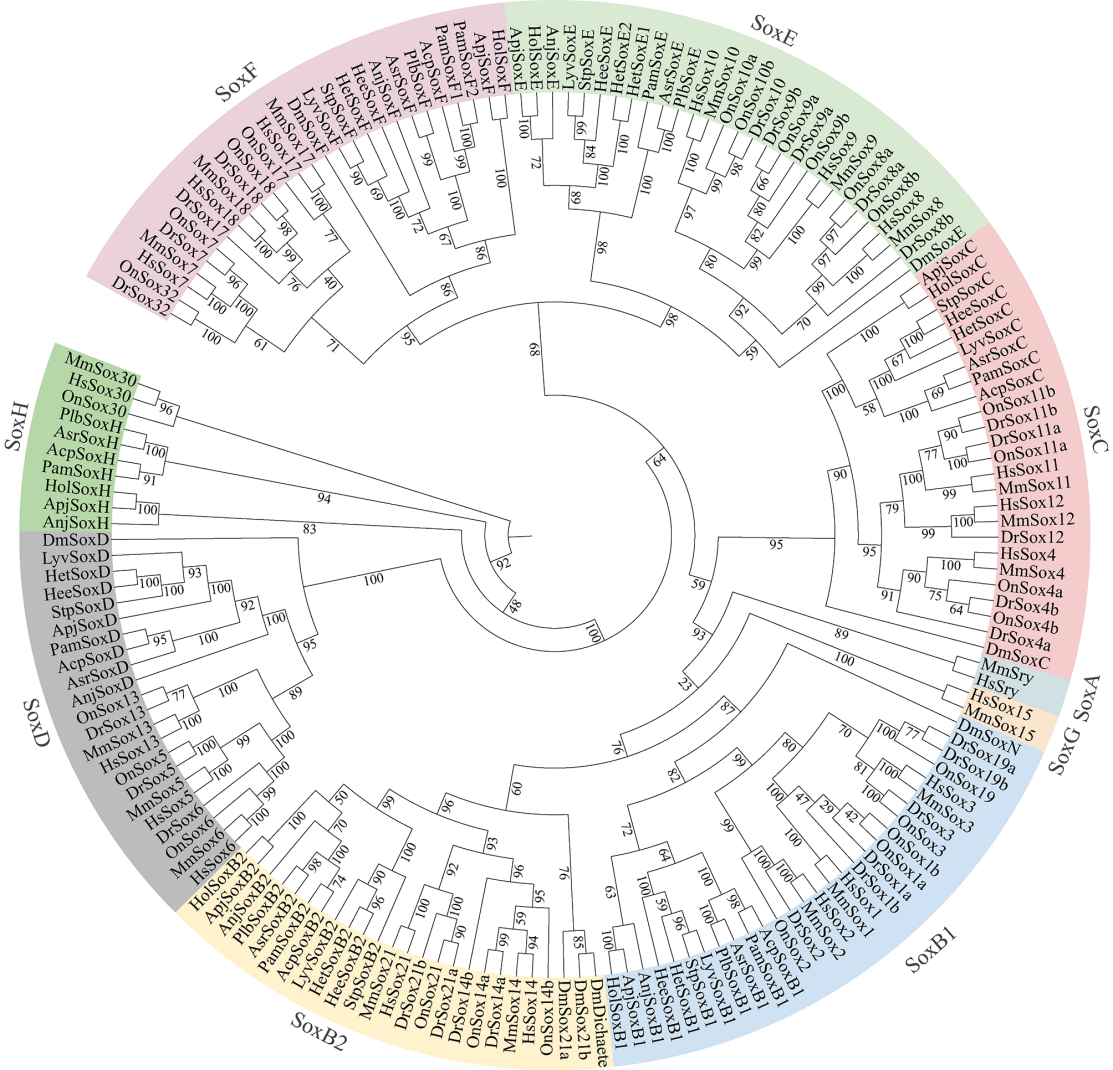
Phylogenetic tree of SOX protein sequences

A phylogenetic tree including Sox protein sequences obtained from both vertebrates and invertebrates was constructed to investigate the evolutionary relationships among *Sox* genes in echinoderms. As shown in Fig. 1, the 70 *Sox* genes from echinoderms were divided into 7 classes: the *SoxB1* class, *SoxB2* class, *SoxC* class, *SoxD* class, *SoxE* class, *SoxF* class and *SoxH* class. Within the *SoxB1*, *SoxB2*, and *SoxF* classes, 11 genes were identified from 11 echinoderms. The *SoxC* class comprises 9 *Sox* genes derived from 9 echinoderms, excluding *A. japonica* and *P. borealis*. Similarly, a *SoxD* class was formed by 9 *Sox* genes from 9 echinoderms. Additionally, the *SoxE* class included 11 *Sox* genes isolated from 10 echinoderms. Notably, *Sox* genes belonging to the *SoxH* class were specifically identified in sea cucumbers and starfish.

### Gene structures and conserved motifs of *Sox* genes

**Fig. 2:**
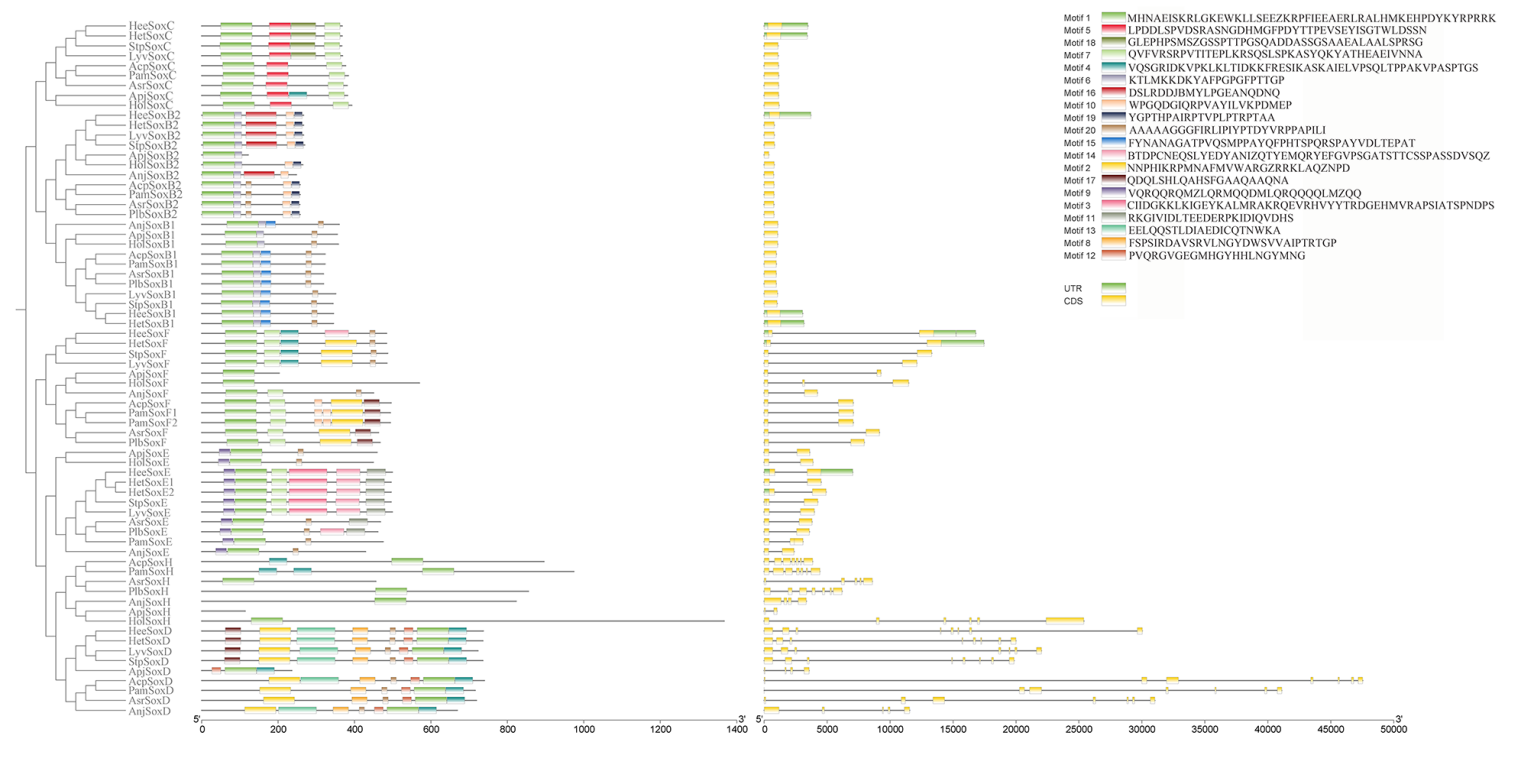
Motif composition and exon‒intron structures of echinoderm *Sox* genes

**Fig. 3:**
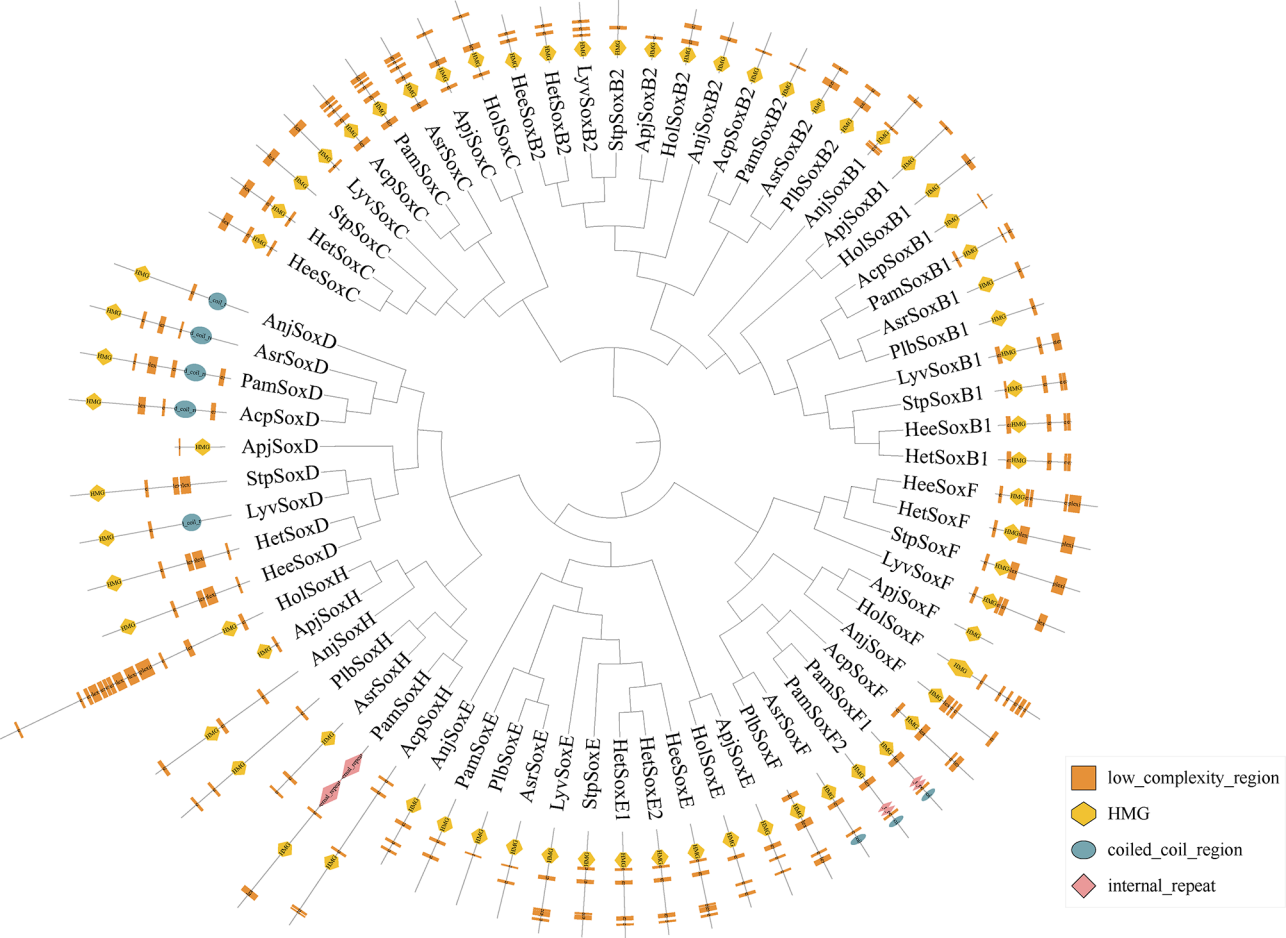
Conserved domain structures of *Sox* genes

The exon‒intron variation among the *Sox* genes of echinoderms is distinctly portrayed in Fig. 2. The exon counts of the *Sox* genes, which varied from 1 to 8 within the same class of 11 echinoderms, displayed a significant degree of similarity in their exon‒intron configurations. This observation suggested the existence of conserved patterns among distinct subsets of the *Sox* family. Furthermore, all the predicted Sox proteins contained motif 1, which suggests that this motif is a common functional element shared by members of this gene family. Proteins within the same class also display greater similarity in their motif structural features, further highlighting the conservation of specific characteristics among related Sox proteins. Additionally, some *Sox* genes were found to contain coiled-coil regions, low-complexity regions, and internal repeats (Fig. 3).

### Spatiotemporal expression of sox genes in three echinoderms

**Fig. 4:**
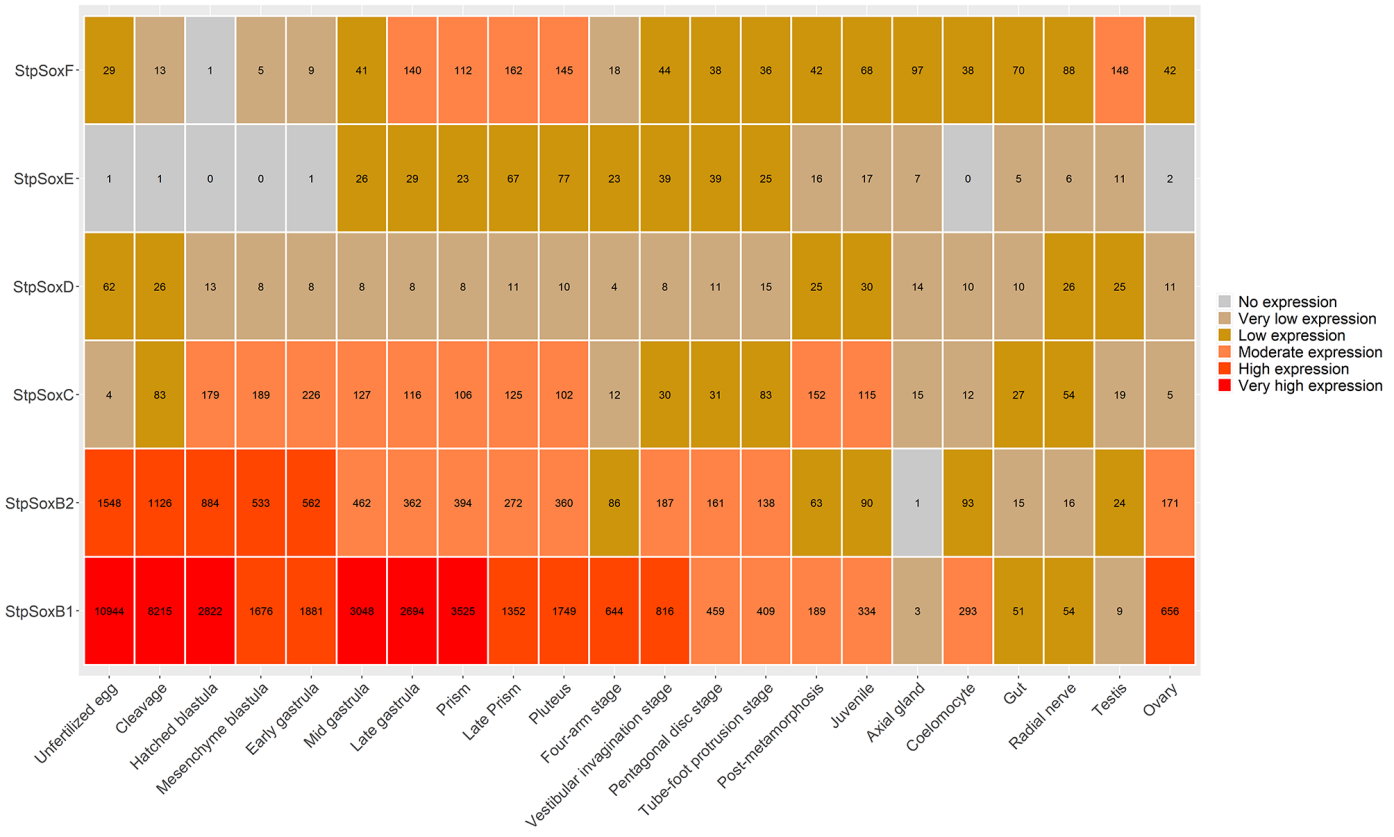
*Sox* gene expression patterns at different developmental stages and in different adult tissues of *Strongylocentrotus purpuratus*

RNA-seq datasets from different developmental stages and adult tissues of *S. purpuratus* were analyzed to investigate the expression patterns of different *Sox* genes. As illustrated in Fig. 4, the expression level of *SoxB1* was particularly prominent during the early development stage, after which it gradually decreased from the initial stage of unfertilized eggs. *SoxB2* exhibited a similar expression pattern to *SoxB1*, albeit with a reduced expression level at the corresponding developmental stage. The expression of *SoxC* initially increased, followed by a subsequent decrease, beginning at the unfertilized egg stage. Conversely, *SoxD* and *SoxE* maintained low expression levels during the early developmental stages. Notably, the primary expression phase of *SoxF* occurred between late gastrula and the pluteus. These results were verified at the single-cell level (Figure S1). For example, the expression of *SoxB1* was prominent across specific clusters (7–10) from early time points, including the 8-cell stage to the early blastula stage (Figure S2), which is very similar to that previously reported for *SoxB2* [28]. In addition, *SoxC* was rarely expressed during the 8-cell stage and 64-cell stage but later exhibited expanded expression across different clusters from the morula stage (Figure S3). Furthermore, at the adult stage, *SoxB1* and *SoxB2* exhibited the highest expression levels in the ovary, whereas *SoxF* demonstrated peak expression in the testis. Additionally, *SoxC*, *SoxD*, and *SoxE* exhibited consistently low expression across all tissues examined.

**Fig. 5:**
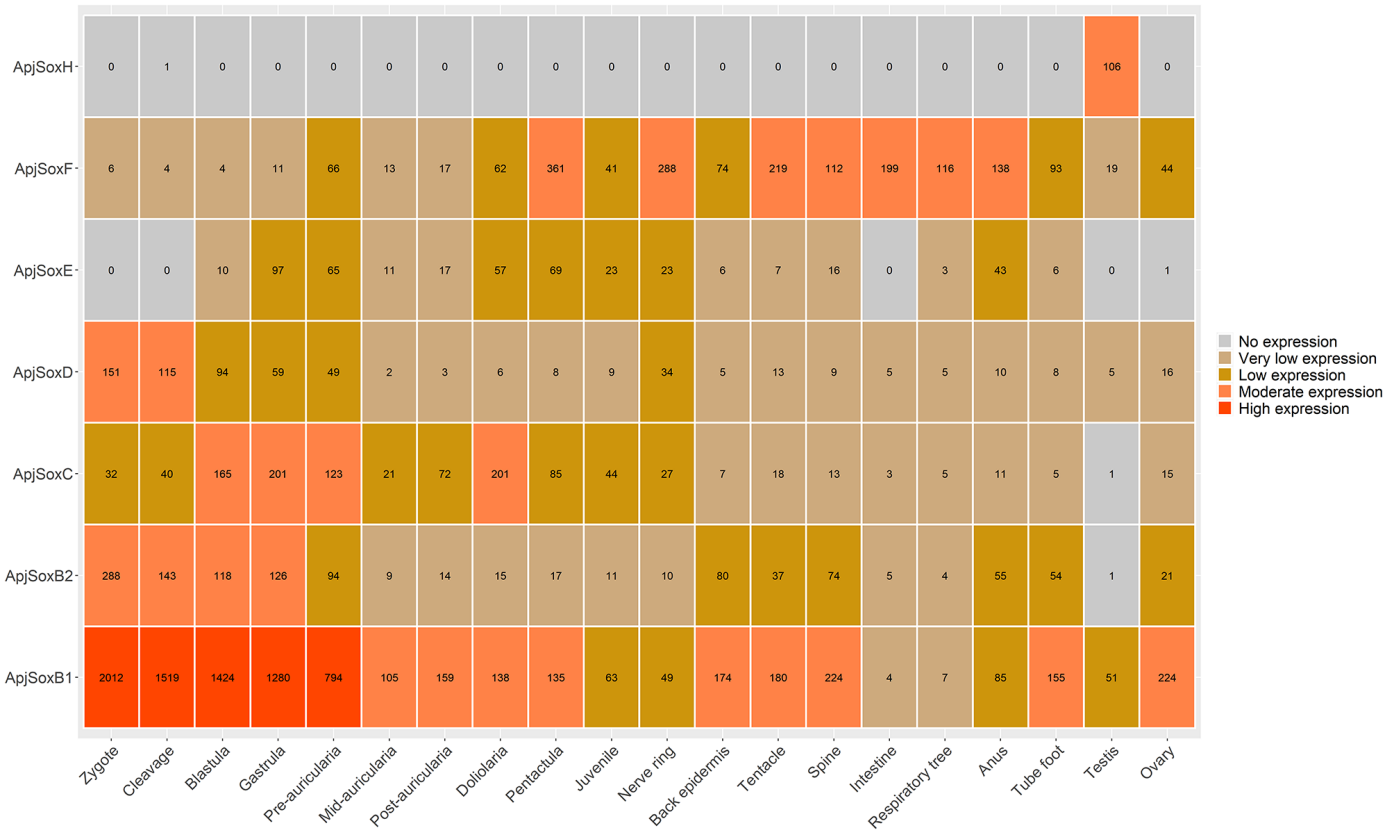
*Sox* gene expression patterns at different developmental stages and in different adult tissues of *Apostichopus japonicus*

In *A. japonicus* (as depicted in Fig. 5), *SoxB1*, *SoxB2*, and *SoxD* exhibited similar expression trends. However, notably, the expression levels of *SoxB2* and *SoxD* were significantly lower than those of *SoxB1*. Additionally, the expression of *SoxC* was particularly pronounced during the gastrula and doliolaria stages, surpassing its expression in other developmental stages. On the other hand, *SoxF* demonstrated a greater expression level specifically in the pentactula stage than in the other developmental stages. It was also observed that the expression of *Sox* genes decreased gradually from the initial stage in unfertilized eggs. At the adult stage, *SoxB1* presented the highest expression in the ovary and spine, but *SoxB2* and *SoxF* exhibited the highest expression levels in the back epidermis and nerve ring, respectively. Intriguingly, *SoxH* displayed high expression specifically in the testis, with no detectable expression in other tissues. Furthermore, *SoxC*, *SoxD*, and *SoxE* maintained consistently low expression levels across all tissues examined.

**Fig. 6:**
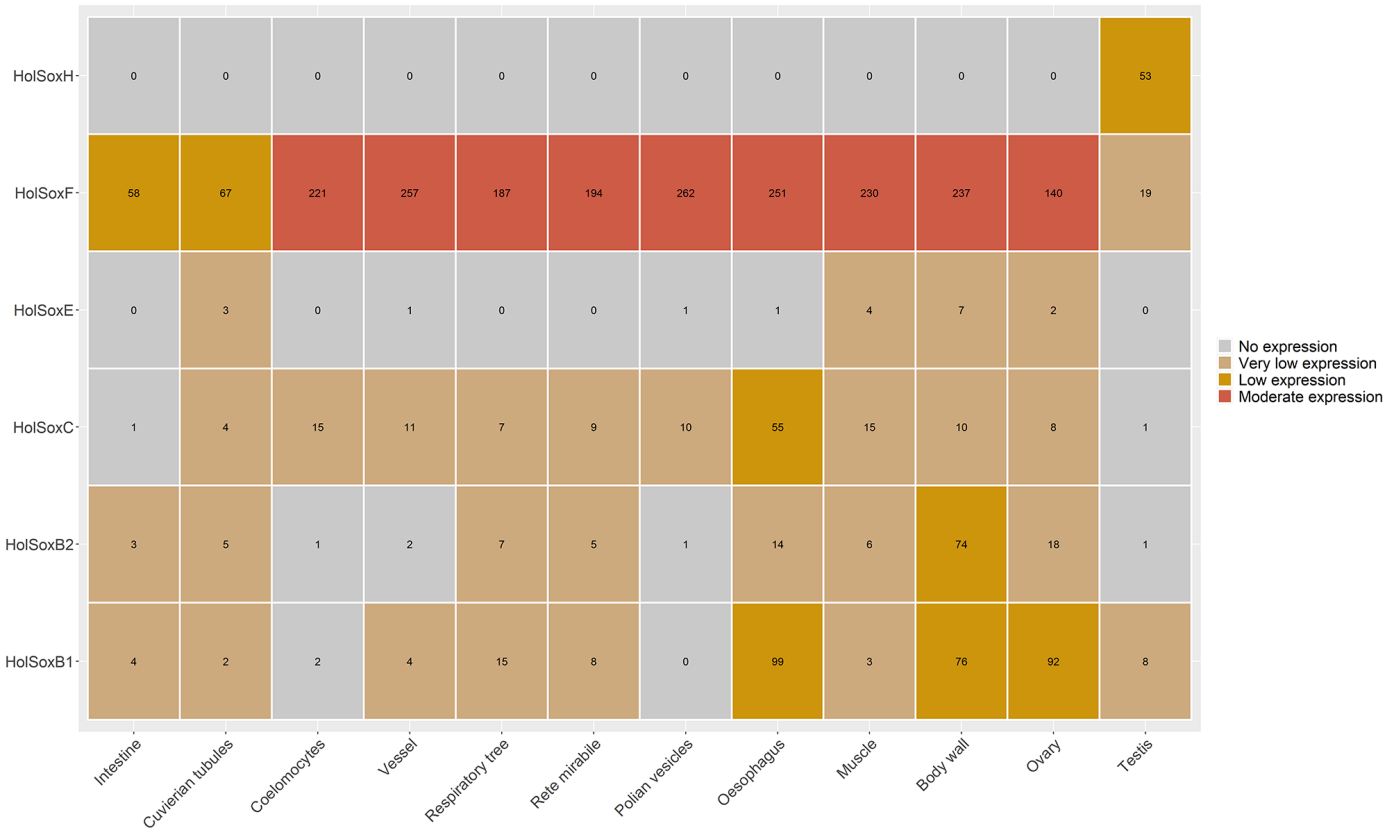
*Sox* gene expression patterns in different adult tissues of *Holothuria leucospilota*

The analysis of *Sox* gene expression across various tissues of *H. leucospilota* revealed distinct patterns (Fig. 6). Specifically, *SoxB1*, *SoxB2*, *SoxC* and *SoxE* exhibited low or even no expression across all tissues examined. Conversely, *SoxF* exhibited a widespread moderate expression pattern and was prominently expressed in multiple tissues, such as coelomocytes, vessels, respiratory trees, rete mirabile, polian vesicles, muscle, body walls, and ovaries. Additionally, *SoxH* was significantly expressed specifically in the testis.

## Discussion

Comprehensive identification of *Sox* family genes has been conducted across diverse animal groups [29–31]. However, the understanding of *Sox* genes in aquatic invertebrates remains limited. Despite the availability of echinoderm genome sequences for several years, a thorough investigation of *Sox* genes in these organisms has yet to be undertaken. In the present study, a comprehensive analysis of *Sox* genes was carried out across different echinoderms. This investigation revealed the presence of five to seven *Sox* genes across different echinoderms. Based on previous research, the variation observed in the number of *Sox* family genes can be ascribed to disparities in genome size and the occurrence rate of genomic duplication events [32]. Despite their ubiquitous presence across the animal kingdom, *Sox* genes exhibit unique species-specific traits. For example, *SoxA* and *SoxG* are exclusively found in mammals. This pattern was confirmed in this study. No *SoxA* or *SoxG* genes were detected in echinoderms, while the *SoxB*, *SoxC*, *SoxD*, *SoxE*, *SoxF* and *SoxH* genes were detected in this study. Similar results were reported in a previous study in which a single C, D, E, F and H and two SoxB proteins were identified in *Ciona* [28]. In addition, the *SoxB1*, *SoxB2* and *SoxF* genes were found in all the studied echinoderms, which suggested that these genes may be conserved in echinoderms.

Unlike certain fish species that possess four *SoxB1* paralogs (*Sox1a*, *Sox1b*, *Sox2*, and *Sox3*), all echinoderms investigated in this study exhibited a single *SoxB1* gene, suggesting that the *SoxB1* gene is conserved within the echinoderm lineage. Previous research has demonstrated that the *SoxB1* gene plays a pivotal role in the initial stages of embryonic development [33–35]. In particular, *SoxB1* has been proven to be a crucial factor in the initiation of the zygotic developmental program [36]. In this study, the *ApjSoxB1* gene was highly expressed in the zygote stage of sea cucumbers. These results indicated that *SoxB1* may perform similar functions in echinoderms. Notably, the expression level of *SoxB1* in the ovaries of the three echinoderms was significantly greater than that in the testis. Similar results have been reported for *L. vannamei* [37]. Thus, it would be intriguing to explore whether *SoxB1* plays important roles in the oogenesis of echinoderms.

It is believed that the *SoxB1* and *SoxB2* genes emerged through the tandem duplication of a genomic segment harboring the putative ancestral *SoxB* gene [38]. *SoxB2* has been extensively studied in invertebrates. For instance, the *SoxB2* gene plays a crucial role in the maturation of the sperm nucleus in *Eriocheir sinensis* [39]. A similar function can also be found in scallops [40]. In contrast, the present investigation revealed that *SoxB2* gene expression was absent or present at minimal levels in the testes of the three echinoderms examined. In addition, previous studies showed that *SoxB2* can function in neurogenesis, ciliogenesis and skeletal patterning in sea urchins [41]. These results suggested that *SoxB2* may have different functions in echinoderms. Furthermore, in this study, the spatial and temporal expression patterns of *SoxB2* in sea cucumbers were very similar to those in sea urchins. Therefore, *SoxB2* may have a conserved function in echinoderms.

In mammals, *SoxC* genes participate in neural and mesenchymal progenitor cell survival, in part by activating this transcriptional intermediary of the Hippo signaling pathway [42]. Although *SoxC* has been identified in some aquatic invertebrates, little research has investigated its function. A previous study showed that *SoxB2* and *SoxC* orthologs play a consistent role in the early neural specification of sea urchins [43]. In this study, the expression of *SoxC* genes in different echinoderms was similar in early development, and the expression level was the highest in the nerve tissue of sea cucumber and sea urchin at the adult stage. Therefore, *SoxC* genes may play a conserved neurogenic role in echinoderms. Similarly, *SoxD* and *SoxE* have been shown to be involved in neurodevelopmental processes [44–47]. However, in the present study, the expression patterns of *SoxD* and *SoxE* were different from that of *SoxC*. Therefore, the functions of *SoxD* and *SoxE* in echinoderms need further study.

*SoxH* genes, which were previously thought to be mammalian specific, have been identified in several invertebrates, including ascidians [28], oysters [48], clams [49], scallops [9], and abalone [50]. *SoxH* usually has male-biased expression in these mollusks. The current study revealed comparable expression patterns in both *H. leucospilota* and *A. japonicus*, indicating that *SoxH* may play a role in determining or facilitating male sexual development in starfish and sea cucumber. Furthermore, these observations suggest that the function of *SoxH* may be conserved across invertebrates and vertebrates. In vertebrates, *SoxF* has been found to be associated with vascular development [51, 52]. In addition, *SoxF* is part of a negative feedback loop in the wingless pathway that controls proliferation in Drosophila wing discs [53]. However, to date, the function of the *SoxF* gene in aquatic invertebrates remains unclear. In the present study, *SoxF* was ubiquitously expressed in early developmental stage and adult tissues. A similar result was found in the Pacific abalone *Haliotis discus hannai* [50]. These results suggest that the *SoxF* gene has diverse functions in aquatic invertebrates. In general, the present investigation provides a molecular foundation for exploring the *Sox* gene in echinoderms, providing a valuable resource for future phylogenetic and genomic studies.

## Conclusion

In this study, a systematic analysis of *Sox* family genes in 11 echinoderms was performed. A total of 70 *Sox* genes were found, and the number of *Sox* genes in different echinoderms ranged from 5 to 8. All *Sox* genes from echinoderms were classified into 7 classes: the *SoxB1* class, *SoxB2* class, *SoxC* class, *SoxD* class, *SoxE* class, *SoxF* class and *SoxH* class. Furthermore, the spatiotemporal expression of *Sox* genes from three echinoderms suggested that different *Sox* family members have different functions. Notably, *SoxH* may play a crucial role in the testis development of starfish and sea cucumber, while *SoxB1* is likely involved in echinoderm ovary development. In general, the present investigation provides a molecular foundation for exploring the *Sox* gene in echinoderms, providing a valuable resource for future phylogenetic and genomic studies.

### Electronic supplementary material

Below is the link to the electronic supplementary material.

Supplementary Material 1

Supplementary Material 2

Supplementary Material 3

Supplementary Material 4

Supplementary Material 5

## Data Availability

The datasets generated and/or analyzed during the current study are available in the NCBI [GCA_001949145.1, GCA_011630105.1, GCA_002754855.1, GCA_902459465.3, GCA_025617745.1, GCA_025618425.1, GCA_029531755.1, GCA_018143015.1, GCA_015706575.1, GCA_021014325.1, GCA_000002235.4, PRJNA81157, PRJNA413998, PRJNA646282, and PRJNA747844].
