# Supplementary figures and images for: Identification and expression analysis of Sox family genes in echinoderms
Source: BMC Genomics. 2024 Jul 1;25:655. doi: 10.1186/s12864-024-10547-0 (PMC11218330; doi:10.1186/s12864-024-10547-0)

Figure S1


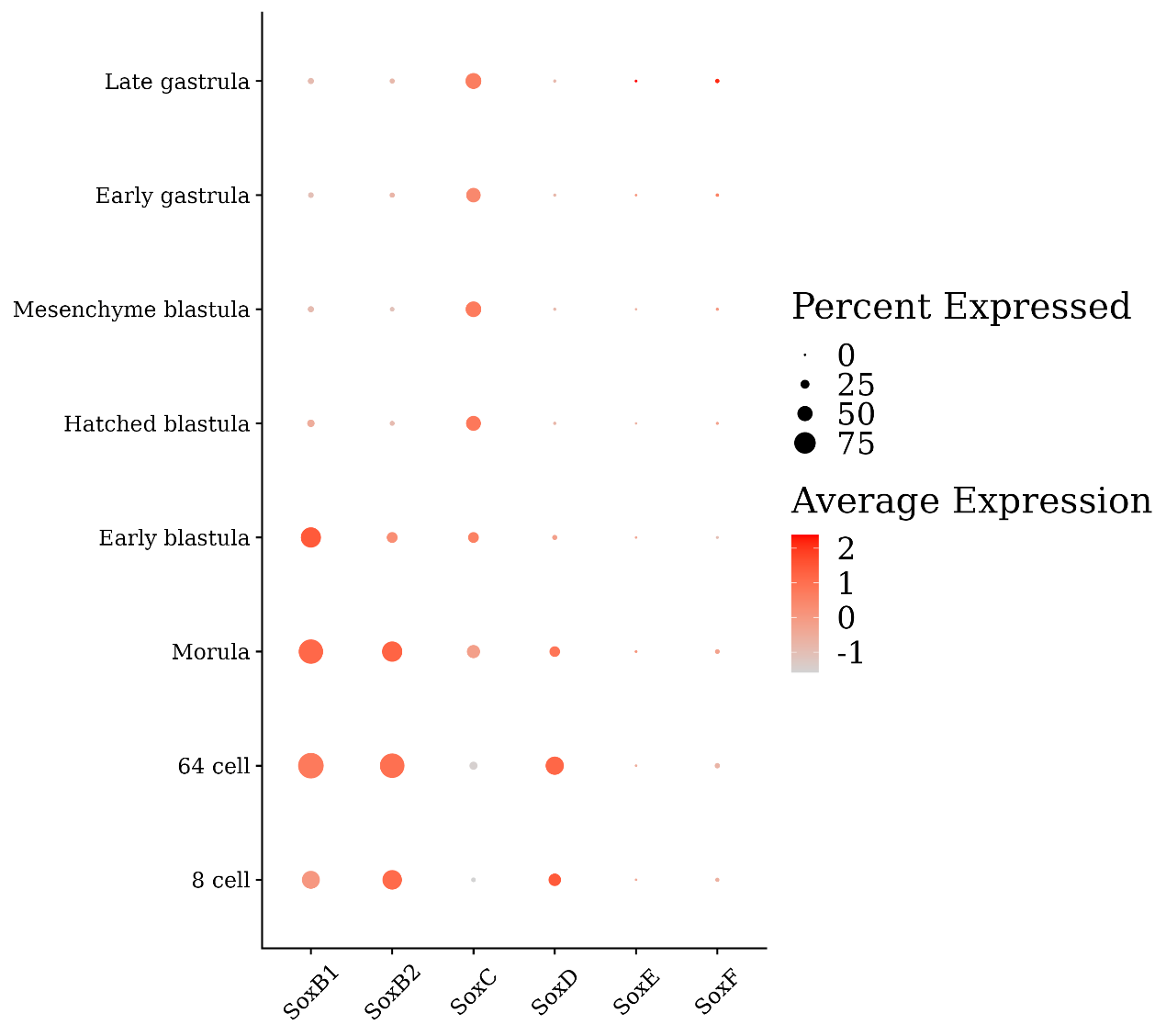


Figure S2


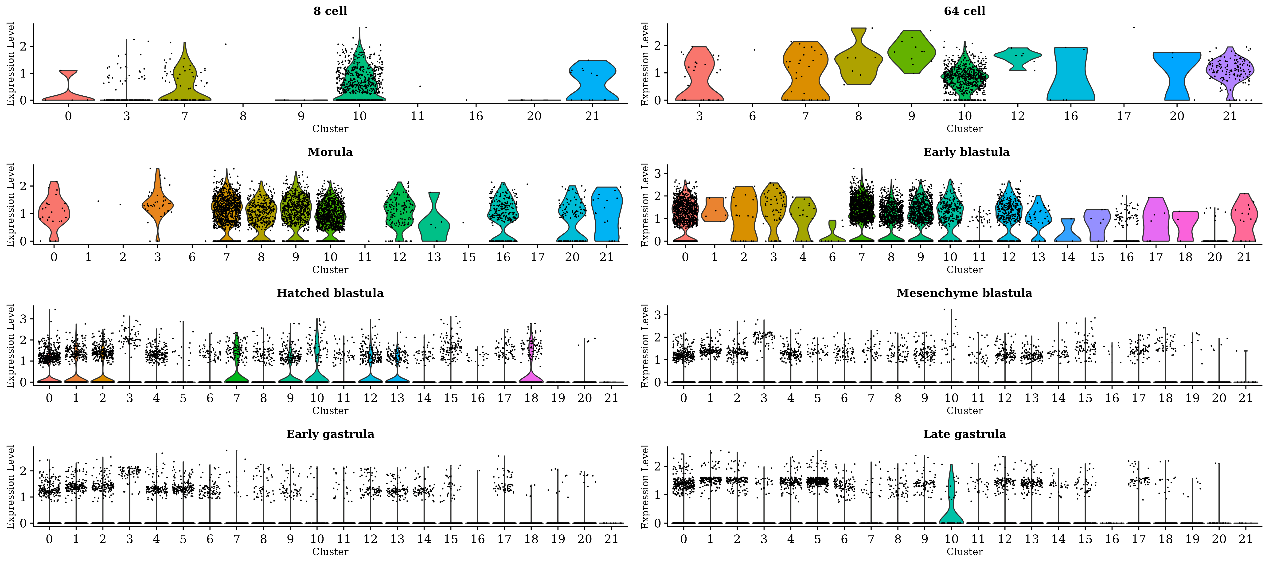


Figure S3


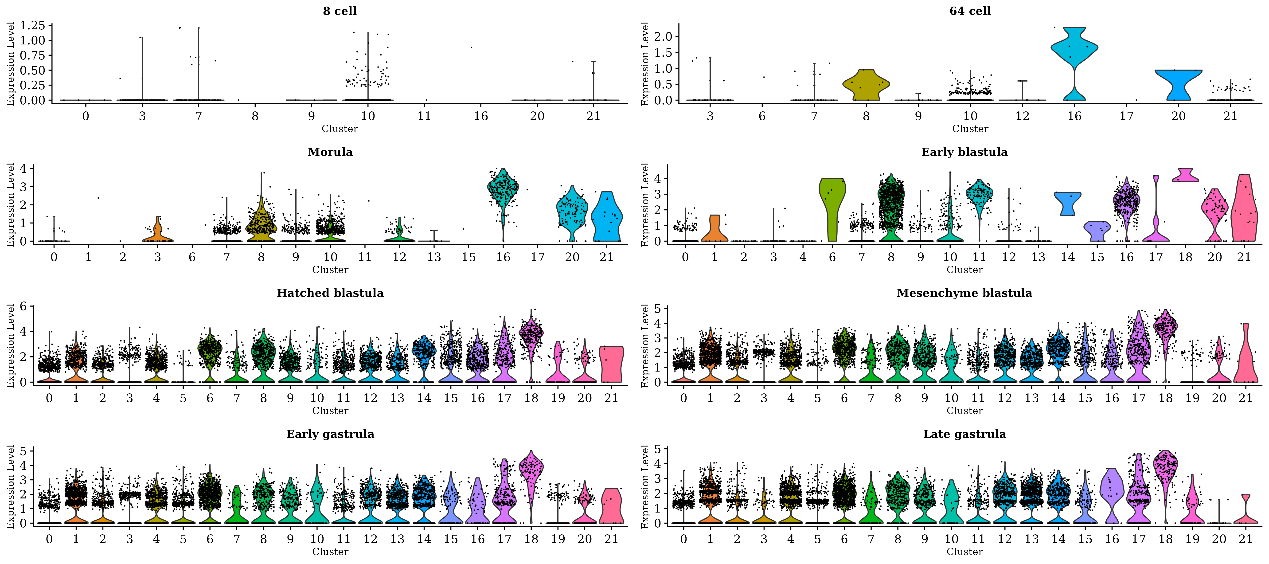

Supplement: Supplementary file 1 — Supplementary Material 1 [file 12864_2024_10547_MOESM1_ESM.doc]

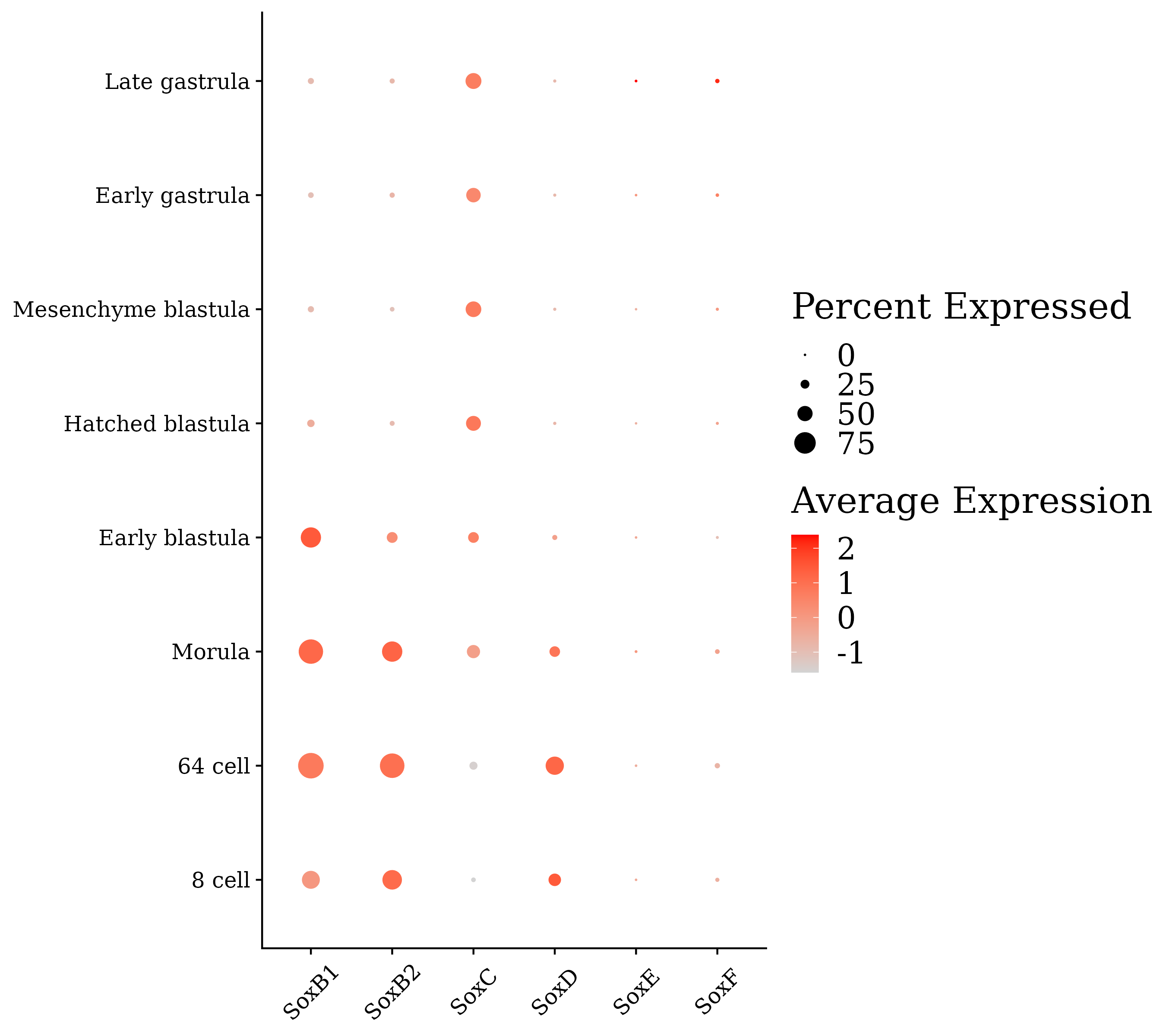

Supplement: Supplementary file 3 — Supplementary Material 3 [file 12864_2024_10547_MOESM3_ESM.tif]

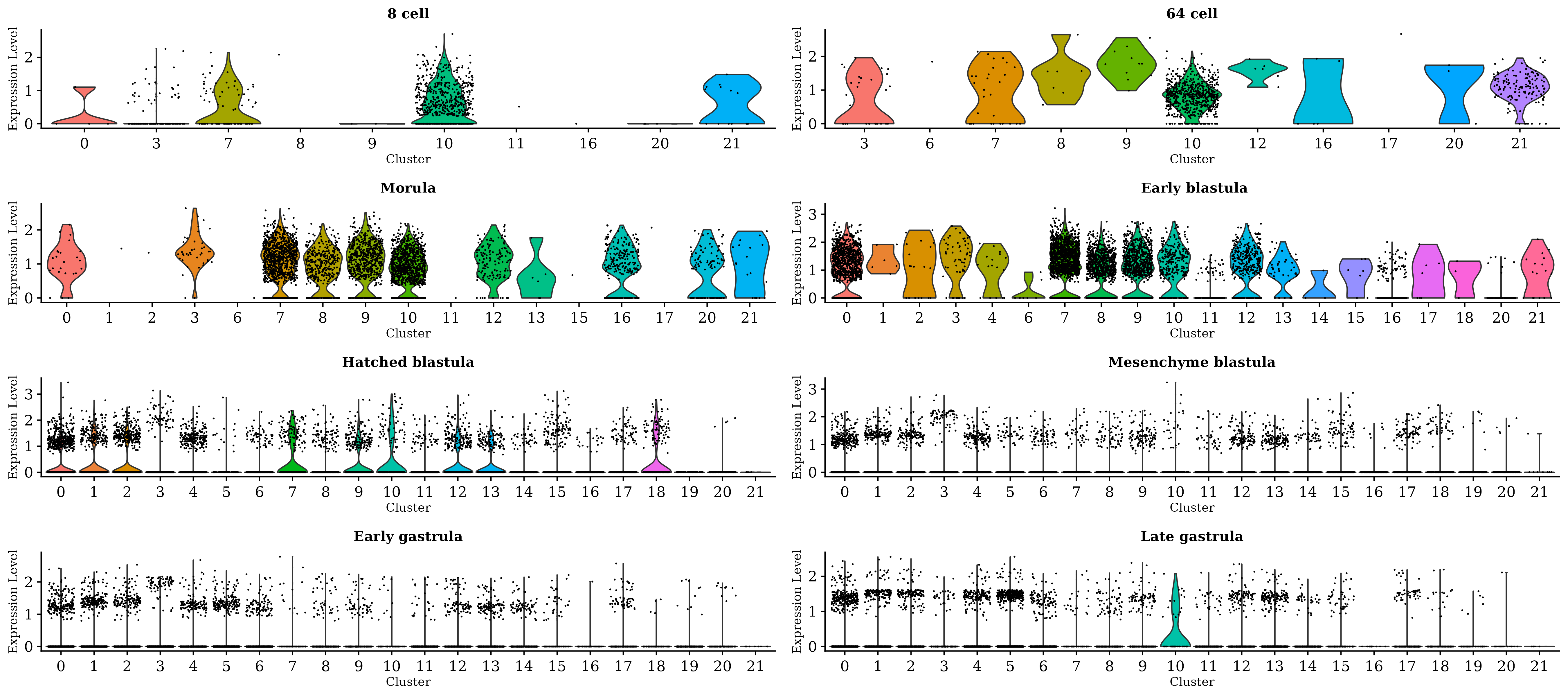

Supplement: Supplementary file 4 — Supplementary Material 4 [file 12864_2024_10547_MOESM4_ESM.tif]

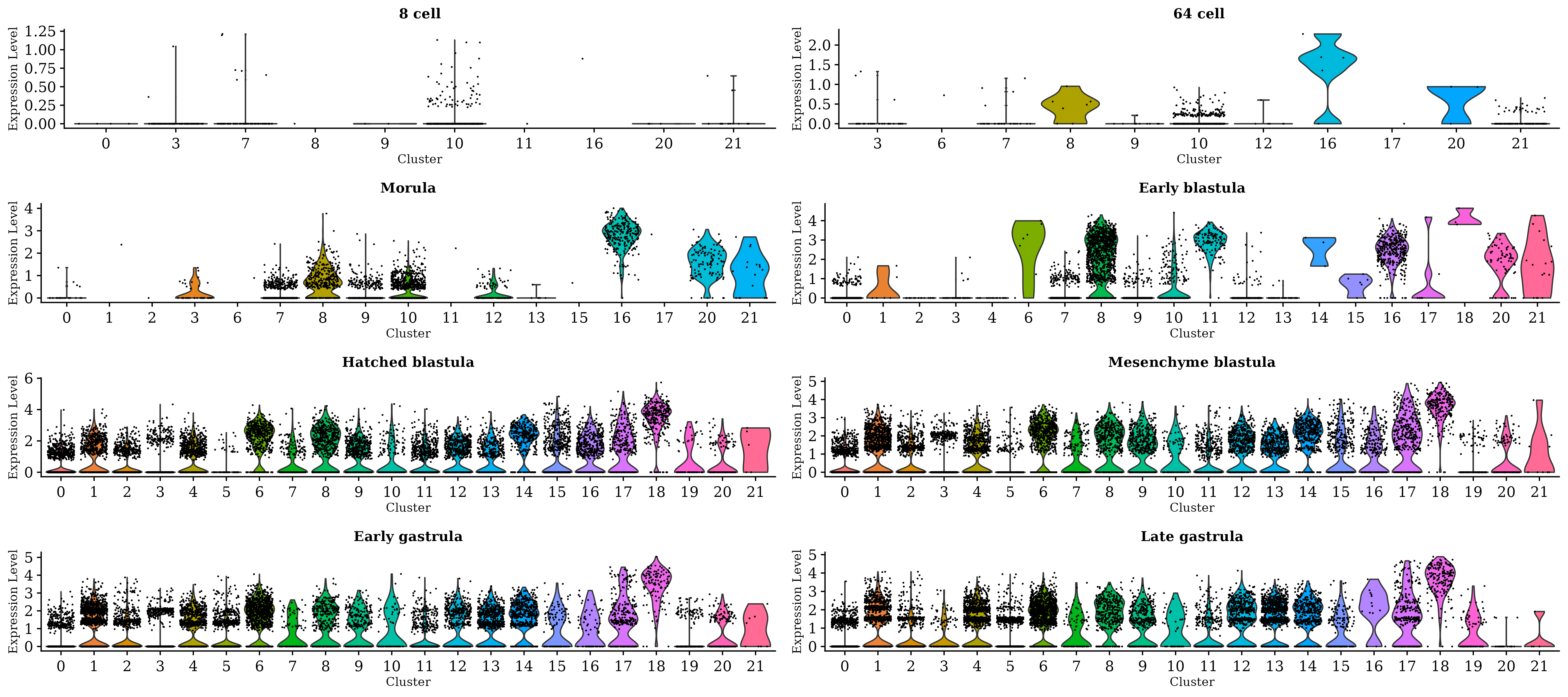

Supplement: Supplementary file 5 — Supplementary Material 5 [file 12864_2024_10547_MOESM5_ESM.tif]
